# Whey Proteins and Metabolic Dysfunction-Associated Steatotic Liver Disease Features: Evolving the Current Knowledge and Future Trends

**DOI:** 10.3390/metabo15080516

**Published:** 2025-08-01

**Authors:** Maja Milanović, Nataša Milošević, Maja Ružić, Ludovico Abenavoli, Nataša Milić

**Affiliations:** 1 Department of Pharmacy, Faculty of Medicine, University of Novi Sad, Hajduk Veljkova 3, 21000 Novi Sad, Serbia; maja.milanovic@mf.uns.ac.rs (M.M.); natasa.milic@mf.uns.ac.rs (N.M.); 2 Faculty of Medicine, University of Novi Sad, Clinic for Infectious Diseases, University Clinical Centre of Vojvodina, Hajduk Veljkova 1, 21000 Novi Sad, Serbia; maja.ruzic@mf.uns.ac.rs; 3 Department of Health Sciences, University “Magna Graecia”, 88100 Catanzaro, Italy; l.abenavoli@unicz.it

**Keywords:** obesity, body composition, bioactive food compounds, metabolic disorders, diet, fatty liver

## Abstract

Metabolic dysfunction-associated steatotic liver disease (MASLD), previously known as non-alcoholic fatty liver disease (NAFLD), is a prevalent, multisystem disease affecting approximately 30% of adults worldwide. Obesity, along with dyslipidemia, type 2 diabetes mellitus, and hypertension, are closely intertwined with MASLD. In people with obesity, MASLD prevalence is estimated to be about 75%. Despite various approaches to MASLD treatment, dietary changes remain the most accessible and safe interventions in MASLD, especially in obese and overweight patients. Whey proteins are rich in bioactive compounds, essential amino acids with antioxidant properties, offering potential benefits for MASLD prevention and management. This state-of-the-art review summarizes whey protein impacts on a spectrum of MASLD-related manifestations, such as obesity, impaired glucose and lipid metabolism, hypertension, liver injury, oxidative stress, and inflammation. The results obtained in clinical environments, with a focus on meta-analysis, propose whey protein supplementation as a promising strategy aimed at managing multifaced MASLD disorders. Well-designed cohort studies are needed for validation of the efficacy and long-term safety of whey proteins in MASLD patients.

## 1. Introduction

Metabolic dysfunction-associated steatotic liver disease (MASLD) is the most prevalent chronic liver disease worldwide, affecting approximately 38% of the global adult population and rising to an estimated 75% among individuals with obesity [1,2]. MASLD, previously known as non-alcoholic fatty liver disease (NAFLD) and as metabolic dysfunction-associated fatty liver disease (MAFLD), is strongly associated not only with obesity, but also with type 2 diabetes mellitus (DM), hypertension, and other metabolic risk factors [3]. The disease can progress from hepatic steatosis to metabolic dysfunction-associated steatohepatitis (MASH), potentially advancing through stages of fibrosis, cirrhosis, and ultimately hepatocellular carcinoma (HCC). The severe form, MASH, is expected to increase by over 50% in the next decade across Europe, the USA, and Asia. MASH is one of the fastest-growing causes of hepatocellular carcinoma, with 0.5–2.6% incidence among patients with cirrhosis, and imposes substantial healthcare costs, surpassing USD 100 billion annually in the USA alone [4]. The anticipated MASLD burden may reach USD 1 trillion in the USA and EUR 334 billion in Europe by 2030 [5].

Dietary interventions remain the mandatory approach to MASLD treatment, despite the effectiveness of applied pharmacological treatments. Variations in the pathogenesis of MASLD and the subsequent subtypes affect the efficacy of pharmacological treatments, often leading to limited or inconclusive outcomes for some medications or the benefits of other pharmaceutics being overshadowed by safety concerns [6,7]. Today, the manipulation of gut microbiota is attracting growing interest from researchers as a promising strategy for management of MASLD. Apart from genetic background, gut dysbiosis in MASLD is mostly driven by a mixture of different factors such as poor dietary habits, lack of physical activity, and medication. As a consequence of the specific microbiome signature (a decreased *Firmicutes* spp. to *Bacteroidetes* spp. ratio), the gut–liver axis is impaired and complex MASLD inflammatory mechanisms occur [8]. Hence, the search for safe, effective, and accessible nutritional interventions that offer benefits in MASLD treatment—particularly among obese and overweight patients—remains a critical priority in addressing this growing public health concern.

Whey is a nutrient-rich by-product of the cheese manufacturing process, traditionally considered a form of biowaste. Through appropriate processing technologies, whey can be efficiently transformed into a high-quality protein supplement. Due to its widespread availability and relatively low production costs, whey proteins represent an economically viable and accessible nutritional intervention [9,10]. This cost-effectiveness positions whey proteins as a promising candidate for inclusion into dietary strategies aimed at managing metabolic disorders such as MASLD, particularly in resource-limited settings. Current evidence about the benefits of whey proteins, focusing on all MASLD risk factors, is presented. In this paper, an overview of the effects of whey proteins on obesity, glucose homeostasis, blood pressure, lipid profile, liver enzymes, oxidative stress, and inflammation are summarized. Original research articles, narrative and systematic review papers, as well as meta-analyses published in the English language were searched using the Google Scholar and PubMed databases up to May 2025, with a focus on whey protein effects on MASLD features (obesity, glucose homeostasis, blood pressure, lipid status, liver injury, oxidative stress, and inflammation). The outcomes from the meta-analysis and the results obtained in randomized clinical trials were discussed in order to provide a more precise estimation of the effect of whey protein supplementation. This review offers a novel perspective on the potential role of whey proteins in MASLD management and underscores the need for further clinical research to establish efficacy, optimal dosing, and long-term safety.

## 2. MASLD

MASLD is diagnosed as a steatotic liver disease in conjunction with at least one of five cardiometabolic risk factors: (1) body mass index (BMI) ≥ 25 kg/m^2^ OR waist circumference (WC) > 94 cm (males), 80 cm (female); (2) Fasting serum glucose ≥ 5.6 mmol/L OR 2 h post-load glucose levels ≥ 7.8 mmol/L OR hemoglobin A1c (HbA1c) ≥ 5.7% OR diagnosis of DM OR treatment for DM; (3) Blood pressure ≥ 130/85 mmHg OR specific antihypertension treatment; (4) Plasma triglycerides (TG) ≥ 1.70 mmol/L OR lipid-lowering treatment; (5) Plasma HDL ≤ 1.0 (M) and ≤1.3 mmol/L (F) OR lipid-lowering treatment [11]. According to some authors, plasma high-sensitivity C-reactive protein level (hs-CRP) > 2 mg/L and homeostasis model assessment (HOMA) with insulin resistance score ≥ 2.5 should also be considered as separate metabolic criteria for MASLD [12]. Regardless of their metabolic features, patients are only considered to have MASLD if the hepatic steatosis was not caused by steatogenic drug use (i.e., corticosteroids, amiodarone, and methotrexate) or excessive alcohol consumption (>20 g/d in females or >30 g in males) [11].

There have been numerous attempts to classify patients with MASLD based on clinical characteristics, laboratory findings, and genetic variants (Table 1). Based on the three major contributors to its pathogenesis, MASLD may be classified in one of the following categories: (1) MASLD with a strong hepatic genetic component, (2) MASLD with a strong metabolic component related to hepatic de novo lipogenesis, and (3) MASLD with a strong metabolic component related to adipose tissue dysfunction [13].

In MASLD patients with a strong hepatic genetic component, hepatic lipid content is high. The risk of insulin resistance, type 2 DM, or adiposity is low in these patients. The risk of MASH and fibrosis is moderate to strong, while the risk of cardiovascular disease is governed by obesity abundance. Although the risk of cardiovascular disease seems the lowest in this group, there is higher risk of MASH, cirrhosis, and hepatocellular carcinoma due to genetic risk variants. For these patients, effective pharmacological treatment to decrease hepatic inflammation and fibrosis should usually be the first therapeutic choice [13].

MASLD with a strong metabolic component related to hepatic de novo lipogenesis includes a high intake of glucose, diabetes-associated hyperinsulinemia, and hyperglycemia. This MASLD type is associated with increased insulin resistance and adiposity, severe dyslipidemia, and hyperglycemia, followed by more pronounced risk for cardiovascular disease. The risk for MASH or fibrosis is moderate. In these patients, the greatest focus of interventions should be on tailoring dietary patterns and exercise recommendations [13].

MASLD with a pronounced metabolic component related to adipose tissue dysfunction includes lipodystrophy, increased visceral fat, reduced gluteofemoral fat, insulin-resistant adipose tissue, increased lipolysis, chronic inflammation, and dysregulated adipokines. The risk for insulin resistance, type 2 DM, and cardiovascular disease is high in this MASLD category, followed by moderate risk of MASH and fibrosis, while low amounts of adiposity and moderate dyslipidemia are observed. Individuals with this MASLD type, particularly those with lean MASLD, may be candidates for pharmacological treatment options rather than diet modification, increased exercise, and weight reduction [13].

Nevertheless, despite MASLD classification attempts, major clinical guidelines consistently include dietary weight loss in all overweight or obese MASLD patients. A reduction in hepatic lipid content is typically observed following a weight loss of at least 5%. Improvements in hepatic inflammation are expected with a weight loss of 7–10%, while regression of liver fibrosis generally requires weight loss exceeding 10% [14]. Even among lean MASLD individuals, despite the pharmacological options, the weight loss should aim for a targeted 3–5% [15].

## 3. Whey Proteins

Whey is a by-product of the dairy industry, generated during the production of cheese and casein. Whey is obtained by casein coagulation of any milk type using proteolytic enzymes of animal and microbiological origin (“sweet whey”) or acid (“sour whey”) and accounts for around 90% of the total amount of milk that is transformed into cheese [16,17]. Based on global estimation, almost 200 million tons of whey are produced annually, and half of this amount originated from the cheese industry [18].

Whey contains about 50% of milk components of a high nutritional value and its composition is strongly related to the milk type and animal diet, as well as production processes and environmental factors [16,19]. Almost 95% of fresh whey is water, and after evaporation, the total solid comprises lactose (70–80% of dry matter), proteins (around 9% of dry matter), fats (up to 1%), minerals, and water-soluble vitamins [20]. Although present in relatively small amounts, the lipids in whey might act as a carrier for fat-soluble compounds, whereas folic acid, B12, and niacin, together with minerals (calcium, magnesium, sodium, potassium, phosphate) that are responsible for osmotic balance maintenance and neuro-muscular function, are present in whey in soluble form [21].

Whey proteins, particularly α- and β-lactalbumins that present in an approximate ratio of 1:3, are the most valuable whey constituents. In addition, serum albumin, immunoglobulins, glycomacropeptide, lactoferrin, and lactoperoxidase are part of the whey protein fraction, together with casein degradation products such as proteose-peptone components and growth factors [16,22]. Whey protein composition is also related to the type of milk, whey processing (“sweet whey” or “sour whey”), animal lactation stage, and diet of the dairy animal. These variables can affect both the qualitative and quantitative profile of whey proteins, thereby impacting their nutritional and functional properties [16]. Whey proteins are rich in sulfur-amino acids (methionine and cystine) and exhibit a favorable ratio of essential amino acids (isoleucine, lysine, threonine, and tryptophan) that have crucial role in preventing oxidative stress and inflammation, as well as in maintaining lipid metabolism and glucose homeostasis [17,23].

Although whey was used in ancient times to enhance vitality and as a medicine [22], until recent decades, it was predominantly considered a waste product. Despite its high nutritional value, whey has often been discharged as wastewater, posing a significant environmental threat due to the high content of organic load and high biochemical and chemical oxygen demand (BOD = 35–45 g/L and COD = 50–80 g/L, respectively) [24]. Therefore, numerous procedures and technologies have been developed worldwide for whey utilization, aiming to protect the environment and valorize nutritionally valuable constituents. Whey is commonly used in animal nutrition, as a fertilizer and plant nutrient, but also in the food industry as a component of infant formulas, as well as in the production of functional food and supplements, due to the observed beneficial effects on human health [25]. The most important whey products are whey powder (dehydrated whey), whey protein concentrates (up to 80% of protein), whey protein isolate (≥90% protein), lactose, single protein fractions (i.e., β-lactalbumins, lactoferrins, imunoglobulins), and protein hydrolysates [26]. With the increasing global trend in healthier lifestyles, the consumption of whey protein powders and isolates has significantly increased. This growing popularity has been largely attributed to the excellent bioavailability of whey proteins, which are rapidly digested and absorbed within approximately two hours following ingestion. Their high gastrointestinal absorption efficiency makes them particularly suitable for use in sports nutrition, clinical nutrition, and metabolic disorder management [27]. The large-scale production of whey protein hydrolysates, which are rich source of bioactive peptides, still remains a real challenge, due to the complex globular structure of whey proteins and the high degree of hydrolysis, controlled pH, and temperature conditions required. Moreover, the resulting hydrolysates must undergo rigorous safety and efficacy validation before they can be approved for use in functional foods [28]. Whey protein’s beneficial effects on appetite and weight control have been acknowledged in animal studies [29,30]. In addition, whey protein hydrolysates manufactured from elastase resulted in the inhibition of angiotensin I-converting enzyme (ACE) and pronounced anti-hypertensive effects [31]. Whey bioactive peptides ameliorated inflammation, insulin resistance, lipid accumulation, and oxidative stress in pre-clinical studies [30,32,33,34,35,36,37]. The inhibition of alanine transaminase (ALT) and aspartate transaminase (AST) activity by whey was also observed in vitro in serum with pathological (ALT-10.71%, AST-8.51%) and normal values (ALT-39.33%, AST-29.08) [38]. Oral administration of whey protein isolate in rats resulted in a significant decrement in lipid peroxidation, TG, and liver enzyme (ALT and AST) levels, indicating hepatoprotective and antioxidative effects [29]. Interestingly, a diet enriched with whey protein hydrolysates had beneficial effects on hepatic ischemia-reperfusion injury in rats with nonalcoholic fatty liver [39]. Finally, data about the effects of whey proteins on human gut microbiota remain scarce and limited to pre-clinical studies. An imbalance in gut microbiota can adversely affect liver health through enhanced intestinal permeability, resulting in gut–liver axis malfunction. Damaged intestinal integrity enables bacterial components to enter the bloodstream, and thus aggravates steatosis and inflammatory responses within the liver [40,41,42]. Pre-clinical studies suggests that whey protein supplementation promotes growth in species beneficial for the gut barrier (*Bifidobacterium* spp., *Lactobacillus* spp., *Allobaculum* spp., *Akkermansia muciniphila* and *Bacteroides* spp.) and helps regain gut microbiota balance [43].

**Figure 1:**
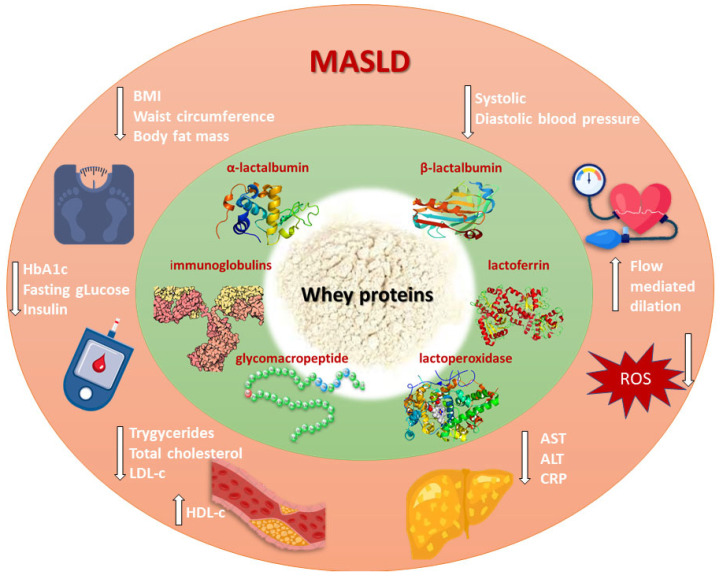
The effects of whey protein bioactive compounds in the management of MASLD. BMI–Body mass index; HbA1c–Hemoglobin A1c; LDL-c–Low-density lipoprotein cholesterol; HDL-c–High-density lipoprotein cholesterol; AST–Aspartate aminotransferase; ALT–Alanine transaminase; CRP–C-reactive protein; ROS–Reactive oxygen species.

In order to verify the effects of whey proteins obtained in pre-clinical studies, the results obtained in humans, with a focus on meta-analyses, will be summarized in the following chapters that address the major MASLD risk factors (Figure 1).

## 4. Whey Proteins and Obesity

The core principle of MASLD pathogenesis is the disruption of energy metabolism balance in the liver. Under physiological conditions, the liver plays a central role in the metabolism of dietary carbohydrates and lipids. Western-style diets, diets high in sucrose, fructose, and saturated fat, or excessive dietary energy intake can overwhelm hepatic metabolic capacity, leading to a net accumulation of energy within the liver in the form of triglycerides. Dietary carbohydrates, predominantly monosaccharides and disaccharides, stimulate de novo lipogenesis and are subsequently converted into hepatic triglycerides, thereby contributing to the progression of MASLD. The high prevalence of MASLD among obese individuals and patients with lipodystrophy is influenced by these metabolic imbalances [44]. Body weight loss of at least 10% may be the single most crucial feature that affects lipogenesis and inhibits the accumulation of intrahepatic triacylglycerols by reducing VLDL-triacylglycerol [45]. In a recently conducted meta-analysis, even modest weight loss was confirmed to significantly improve the clinical features of fatty liver disease. Specifically, for every 1 kg of body weight lost, there was a 0.77 percentage point reduction in hepatic steatosis, as confirmed by radiological or histological assessment (95% CI: 0.51 to 1.03, *p* < 0.0001). Additionally, a dose–response relationship was observed, with progressive weight loss correlating with liver inflammation and hepatocellular ballooning [46]. Whey proteins have been recognized as an effective nutritional supplement capable of reducing body mass and positively affecting body composition. A meta-analysis confirmed that whey protein supplementation for at least four weeks reduced both waist circumference (*p* = 0.03, I^2^: 77%, P_heterogeneity_: 0.00) and body fat mass (*p* = 0.04, I^2^: 00.0%, P_heterogeneity_: 0.44) with statistical significance, followed by borderline significance in BMI decrement (statistically borderline significant (*p* = 0.05, I^2^: 23%, P_heterogeneity_: 0.16)), while preserving lean body mass, since no statistically significant changes in lean body mass were observed between the pre-and post-intervention data. Moreover, the effect of whey protein supplementation was confirmed by comparison between the treatment and control group, as whey protein intake reduced BMI compared with control groups (*p* = 0.05, I^2^: 92.82%, P_heterogeneity_: 0.00), while the decrement in body weight, body fat mass, and waist circumference was not statistically significant. Conversely, the increase in lean body mass observed in the whey protein supplemented group compared to controls was statistically significant (*p* = 0.03, I^2^ = 94.39%, P_heterogeneity_ = 0.00) [47]. These findings are in accordance with a previous meta-analysis, which confirmed that whey proteins not only contributed to both body weight (WGMD: −4.20 kg, 95% confidence interval [CI], −7.67, −0.73) and body fat loss (WGMD: −3.74 kg, 95% CI, −5.98, −1.50), but also statistically significantly increased lean body mass (WGMD: 2.24 kg, 95% CI, 0.66, 3.81) [48]. However, in a separate meta-analysis, whey protein supplementation in obese and overweight individuals significantly reduced body weight (MD = 0.56, 95% CI: 0.30–0.81) and fat mass (MD = 1.12, 95% CI: 0.77–1.47), while unfavorably causing lean mass reduction (MD = 0.77, 95% CI: 0.59–0.96) when compared to controls, with no effects on body fat nor waist circumference [49]. Some later published studies reported contrary findings regarding the effects of whey protein on lean mass. A meta-analysis conducted only on female subjects indicated that whey protein supplementation increased lean mass (WMD, 0.37 kg; 95% confidence interval [CI], 0.06 to 0.67) but had no effect on fat mass (−0.20 kg; 95%CI, −0.67 to 0.27) when compared to the control group [50]. Other studies have also confirmed the beneficial effects of whey proteins on lean mass preservation. A 12-week whey protein intervention compared to a control group resulted not only in significantly greater body fat loss in the treatment group but also in significantly less lean muscle mass loss. Additionally, the treatment group demonstrated an improved fat-to-lean loss ratio (kilograms of fat lost per kilogram of lean mass lost), highlighting a more favorable change in body composition [51]. In contrast, no superior effects of whey protein supplementation in comparison to soy protein nor placebo were reported in a study conducted in Japan. However, a comparison between the postintervention and baseline values confirmed reductions in body weight, body fat, and abdominal circumference, while muscle mass was increased, which is in accordance with previous studies emphasizing the protective effect of whey on lean body mass [52]. Other studies confirmed the effects of whey protein supplementation on separate obesity-related anthropometric features. For example, WC reduction due to whey protein supplementation was confirmed in a meta-analysis conducted on obese and overweight patients (−2.76, 95% CI: 3.83, −1.69; *p* < 0.001, I^2^: 100.0%, P_heterogeneity_: <0.001) [53]. Whey proteins were effective among overweight diabetic patients, as supplementation for 12 weeks led to body weight loss (*p* = 0.0066), BMI decrement (*p* = 0.0058), and WC reduction (*p* = 0.0065), followed by a decline in body fat mass (*p* = 0.0003) measured pre- and post-intervention [54]. Among pre- and mildly hypertensive adults, whey protein supplementation for 12 weeks resulted in body fat, fat percentage, and WC decrements (*p* = 0.010, 0.016, 0.019, respectively) when compared to control [55]. The effects of whey protein supplementation on body composition have also been validated in specific populations, such as older women. Significantly greater reductions in body weight (*p* < 0.05), BMI (*p* < 0.05) and body fat (*p* < 0.01) were observed in older women treated with whey protein supplements for 8 weeks in comparison to controls [56]. In addition, whey protein intake for 12 weeks after resistance training resulted in reducing body fat in older women compared to a placebo group [57].

The bioactive peptides and amino acids produced during gastrointestinal digestion of whey proteins seem to stimulate the secretion of several regulatory hormones, including cholecystokinin (CCK), peptide YY (PYY), glucose-dependent insulinotropic polypeptide (GIP), glucagon-like peptide-1 (GLP-1), and insulin, which may contribute to enhanced satiety and a reduction in energy intake [58].

## 5. Whey Proteins and Hypertension

According to the World Health Organization, more than 1.2 billion of adults worldwide older than 30 years have hypertension, with higher prevalence in low- and middle-income countries [59]. Controversially, almost 50% of the US adult population have hypertension [60]. Owing to this high prevalence, hypertension is considered a major worldwide public health concern.

Hypertension is manifested with increased blood pressure (≥130/80 mmHg), which is commonly followed by heart, brain, and kidneys damage. Despite genetic and environmental factors, hypertension is associated with metabolic disorders. Hypertension, along with elevated serum glucose levels, dyslipidemia, and obesity, constitutes a key component of metabolic syndrome [61]. Hence, hypertension is closely intertwined with MASLD, as both conditions exert reciprocal influences. Hypertension might increase the risk of MASLD onset and development, whereas MASLD, particularly inflammation, DM, and insulin resistance can lead to increase blood pressure. In fact, approximately every second patient with MASLD has hypertension and every other patient with hypertension will develop MASLD [62,63]. In addition, co-existence of MASLD and hypertension increases the risk for fibrosis [61]. Based on a large cohort that included almost 6000 adults, individuals with stage I (OR 1.70, 95% CI: 1.15–2.53) or stage II of hypertension (OR 1.98, 95% CI: 1.38–2.85) had increased risk for significant fibrosis. Moreover, patients with both hypertension and MASLD had higher rates of significant and advanced fibroses (14.83% and 7.47%, respectively) [61].

The antihypertensive properties of whey supplementation were explored in a double-blinded controlled interventional study conducted in the United Kingdom. The effects of whey protein supplementation on blood pressure were evaluated compared to calcium caseinate and maltodextrin as a control in both male and female participants. Forty-two nonsmoking men and women with prehypertension and mild hypertension (120/80–159/99 mmHg), aged 30–77, and with BMI between 20 and 40 kg/m^2^ were included and thirty-eight of them completed the 32-week trial. Whey protein consumption, in a dose of 56 g per day over eight weeks, resulted in a significant reduction in systolic blood pressure (−3.9 mmHg) and diastolic blood pressure (−2.5 mmHg) compared to controls (all *p*-values were 0.05). Moreover, peripheral (−5.7 mmHg), central systolic (−5.4 mmHg), and mean pressures (−3.7 and −4.0 mm Hg) were decreased compared with the maltodextrin control group (*p* = 0.007, *p* = 0.012, *p* = 0.025 and *p* = 0.019, respectively). Whey protein intake, similarly to calcium-caseinate, increased flow mediated dilation by 1.31% compared with controls (*p* < 0.001). Flow mediated dilation is still considered the gold standard for the assessment of endothelial function, reflecting vascular health and early cardiovascular risk [64]. However, whey protein did not cause the significant differences in nitric oxides as mediators of vessel dilation [65]. Although a decrease in ACE activity was not achieved after whey protein intake, significant in vitro inhibition of enzyme activity was observed compared with the controls. It is worth noting that significant reductions in circulatory soluble adhesion molecules, sICAM-1 and sVCAM-1, were detected when compared to controls, which implies that whey protein supplementation might have beneficial effects on both atherosclerosis progression and endothelial dysfunction [65]. Similar findings were obtained in overweight and obese participants of both genders with pre- and mild hypertension. After 12 weeks of intervention, whey protein supplementation (30 g per day) resulted in a borderline significant decrement in systolic blood pressure when compared with maltodextrin as a control (128.2 ± 6.9 mmHg versus 129.5 ± 7.7 mmHg, *p* = 0.052). When the obtained results were stratified by BMI, whey protein anti-hypertensive effects were more pronounced in overweight and obese participants compared to the control group (126.5 ± 6.9 mmHg vs. 128.8 ± 7.4 mmHg, *p* = 0.033), whereas no differences were observed in the normal weight group. In addition, no changes in diastolic blood pressure, endothelium-1, nitric oxide, angiotensin II, or ACE activity were observed between the groups. However, a considerable increment in flow mediated dilation was measured in the whey protein group in contrast to the control group (5.2% versus 0.3%, *p* = 0.040) [55]. The obtained results are additional proof of improvements in endothelial function by whey proteins.

Having in mind that MASLD is more prevalent in men than in women (47% vs. 36%, respectively) [66] and that approximately two-thirds of MASLD patients are obese [2], the antihypertensive effects of whey peptides were also evaluated in forty-five overweight and obese men (BMI = 25–40 kg/m^2^). Following a 12-week intervention, the administration of whey protein concentrates 30 min prior to ad libitum lunch meals resulted in a significant reduction in both systolic (−16.6 mm Hg) and diastolic (−7 mm Hg) blood pressure (*p* = 0.001 and *p* < 0.001, respectively). In addition, the effects of whey protein on blood pressure were more pronounced when compared to soy protein isolate (systolic blood pressure *p* < 0.02; diastolic blood pressure *p* = 0.001) [67]. Sun et al. evaluated the effects of whey protein and whey protein hydrolysate together with an energy-restricted diet in overweight and obese older women. A considerable decrement in systolic blood pressure was measured in the whey protein hydrolysate group after the controlled eight-week weight-loss intervention [56]. On the contrary, no differences in systolic and diastolic blood pressure were observed in overweight/obese patients with type 2 DM after 12 weeks of whey protein isolate intake (ProLYOtin^®^ in a dose of 40 g per day) [54].

In addition, a meta-analysis including eight randomized control trials with a total of 455 participants confirmed whey-protein-related improvements in systolic and diastolic blood pressure in comparison to controls (all *p* values below 0.05), even though the included studies varied in terms of applied dose (20 g/day to 75 g/day) and period of supplementation (from 2 weeks to 15 months) [49]. Similar findings were obtained in another meta-analysis that covered the results obtained in 37 clinical studies with 2344 individuals. Whey supplementation resulted in significantly reduced systolic and diastolic blood pressure when compared to control groups (−7.46, 95% CI: 9.39, −6.13; *p*: <0.001, I^2^: 99.6%, P_heterogeneity_: <0.001 and −5.68, 95% CI: 6.69, −4.67; *p* < 0.001, I^2^: 98.9%, P_heterogeneity_: <0.001, respectively) [53]. However, a pooled analysis of 18 studies with 1177 individuals showed no significant effect of whey protein on diastolic blood pressure, whereas a significant reduction in systolic blood pressure was obtained (−1.54 mmHg; 95% CI: 2.85 to 0.23, *p* = 0.021, I^2^ = 64.2%, *p* < 0.001). The same meta-analysis revealed that a significant decrement in diastolic blood pressure was achieved in studies where whey protein isolate was applied in a dose higher than 30 g/day and in overweight individuals (BMI between 25 and 30 kg/m^2^) with hypertension [68]. A recent meta-analysis that included 31 clinical trials confirmed that whey supplementation decreased both systolic (−2.20 mmHg [−3.89 to −0.51 mmHg]) and diastolic blood pressure (−1.07 mmHg [−1.98 to −0.16 mmHg]). In addition, whey proteins led to a reduction in blood pressure in individuals with hypertension (systolic: −3.95 mmHg [−6.42 to −1.49 mmHg] and diastolic: −1.93 mmHg [−3.50 to −0.36 mmHg]), as well as in overweight/obese individuals (systolic: −3.38 mmHg [−5.96 to to −0.80 mmHg]) [69].

The combined effects of whey protein supplementation and resistance training on cardiometabolic profile were also evaluated in a double-blind, placebo-controlled trial that involved seventy women, 60 years old or more, that were physically independent and without cardiac dysfunction. No significant differences were observed in systolic or diastolic blood pressures before or after the exercise intervention in women that were supplemented with whey protein [57].

Based on the effects obtained in clinical environments, whey proteins are an interesting source of ACE inhibitory peptides that could serve as promising agents in the management and prevention of hypertension [28]. Although β-lactalbumin possess weak ACE-inhibitory activity in its native form, the application of an enzymatic hydrolysis step using pepsin, trypsin, or chymotrypsin could result in more pronounced inhibition. In fact, the peptides LLF and LQKW derived from β-lactalbumin hydrolyzed with thermolysin have demonstrated potent ACE-inhibitory effects in vitro [70]. Further studies are needed in order to investigate the effects of different whey biopeptides in comparison to the frequently prescribed ACE inhibitors, which decrease blood pressure through the inhibition of the ACE enzyme that converts angiotensin I to angiotensin II and acts as a vasoconstrictor [71].

## 6. Whey Proteins and Glucose Metabolism

MASLD and DM are components of metabolic syndrome and are strongly associated with obesity and insulin resistance. Furthermore, MASLD has been identified as a pre-diabetic condition, with patients estimated to have a 2–5 times greater risk of developing type 2 DM [72], while the prevalence of MASLD is 65% among patients with type 2 DM [73]. Insulin resistance represents the primary pathogenic mechanism underlying MASLD and type 2 DM, and its presence is pivotal in the development of both conditions. In the liver, insulin resistance disrupts glucose homeostasis, including increased gluconeogenesis and glycogenolysis, while impairing glycogen synthesis. These alterations contribute directly to the development of hyperglycemia, followed by pancreatic β-cells increasing insulin secretion impaired insulin signaling in skeletal muscle, which results in chronic hyperinsulinemia, culminating in the development of type 2 DM [44]. Obese and overweight patients exhibit abnormal adipose tissue metabolism and peripheral insulin resistance, resulting in a state of lipotoxicity, which is frequently accompanied by hyperglycemia and chronic glucotoxicity, particularly in the presence of prediabetes or type 2 DM [74]. Patients with type 2 DM are at an increased risk of accelerated progression to MASH, advanced hepatic fibrosis, cirrhosis, and hepatocellular carcinoma [75]. Thus, glycose metabolism regulation may be one of the crucial points in the control of MASLD.

Whey protein supplementation in patients with metabolic syndrome was efficient in the regulation of glucose metabolism, resulting in a significant reduction in HbA1c (WMD: −0.15; 95% CI: −0.29, −0.01), insulin (WMD: −0.94; 95% CI: −1.68, −0.21), and HOMA-IR (WMD: −0.20; 95% CI: −0.36, −0.05) in comparison to control in patients with metabolic syndrome and related conditions according to a meta-analysis [76]. These findings are supported by a different meta-analysis that suggested that whey protein supplementation in patients with type 2 DM significantly reduced mean glucose in the area under the curve (standard mean difference [SMD] = −1.295, 95% CI: −1.878 to −0.712, *p* < 0.001), while the mean of insulin (SMD = 0.562, 95% CI: 0.303 to 0.822, *p* < 0.001), gastric inhibitory polypeptide (SMD = 0.349, 95% CI: 0.074 to 0.624, *p* = 0.013), and glucagon-like peptide-1 (SMD = 0.439, 95% CI: 0.154 to 0.724, *p* = 0.003) were increased in comparison to the controls [77]. Whey proteins have demonstrated beneficial effects on glucose metabolism in obese and overweight patients, as according to a meta-analysis, glucose levels were significantly reduced in the whey-protein-supplemented group (MDD 0.76, 95% CI: 0.14–1.38, I^2^ D 99%, P_heterogeneity_ < 0.01) in comparison to controls [49]. These findings are consistent with another meta-analysis on obese and overweight patients with metabolic syndrome, as fasting glucose levels were significantly reduced in the intervention groups compared to control groups (−1.42, 95% CI: 1.52, −1.31; *p* < 0.001; I^2^: 100.0%, P_heterogeneity_ < 0.001) [53]. Among obese women with polycystic ovary syndrome (PCOS), acute (Day 1) and short-term (Day 7) whey supplementation achieved beneficial effects in regulating glucose metabolism, as in OGTT, glucose remained stable (*p* = 0.02) and insulin sensitivity improved (*p* < 0.01) with whey supplementation, which was associated with upregulated glucagon secretion (*p* < 0.01) [78]. The upregulated insulin levels due to whey protein consumption were not followed by an increase in fat mass. This positive effect of whey proteins may be attributed to a high leucine content and to the ability to enhance insulin sensitivity when consumed consistently over periods longer than 12 weeks. Moreover, whey protein supplementation may achieve glycemia control, possibly through the stimulation of incretin hormones, which increase fasting and postprandial insulin release and improve insulin sensitivity [79]. Single dose whey protein in healthy male individuals verified the favorable effects of whey on glucose metabolism. A whey protein feeding trial before OGTT that allowed euglycemia to be maintained throughout a 180 min postprandial period was conducted, and plasma insulin and C-peptide concentrations were increased, followed by a subsequent reduction in free fatty acid (FFA) concentrations. Glucagon concentrations were elevated after protein ingestion. Insulin concentrations were increased by about six times, glucagon approximately fourfold, and C-peptide twice in comparison to the baseline values [80].

## 7. Whey Proteins and Lipid Metabolism

MASLD is closely associated with the excessive accumulation of both triglycerides and cholesterol in the liver [81]. Overweight and obesity act as a trigger linked to reduced insulin sensitivity in adipose tissue. Dysfunctional adipose tissue is marked by enhanced lipolysis—leading to elevated circulating FFAs, reduced adiponectin secretion, and an increased release of proinflammatory adipocytokines. The accumulation of triglycerides within the liver is predominantly influenced by the influx of FFAs into hepatocytes, in a dose-dependent manner [82]. Cholesterol metabolism plays a central role in the pathogenesis and progression of MASLD, while elevated levels of non-high-density to high-density lipoprotein cholesterol ratio are associated with increased risk of hepatic steatosis [83]. The most common lipid profile in MASLD patients is high TG and low HDL-C serum levels [82]. MASLD prevalence was 67% in patients with severe hypertriglyceridemia (≥5.7 mmol/L), and over 30% in patients with moderate hypertriglyceridemia (2.3−5.6 mmol/L) [84]. Among MASLD patients, after obesity and pre-diabetes, low HDL-c levels is the most common feature [85]. Addressing dyslipidemia is mandatory for MASLD patients, due to their increased cardiovascular risk [86].

Long-term whey protein supplementation for over 12 weeks regulated impaired lipid levels according to a meta-analysis. Namely, a decrease in TG serum levels as a result of whey protein supplementation was reported (WMD = −12.21 mg/dL; 95%, CI: −20.16, −4.26; *p* = 0.003), even in patients with obesity, metabolic syndrome, and hypertension in comparison to a placebo. The effects on HDL-c were even more notable, since whey protein intake for less than 12 weeks resulted in a statistically significant elevation in HDL-c (WMD: 2.59 mg/dL; 95%, CI 1.11, 4.07; *p* = 0.001) unlike the placebo, but with no effects on LDL-c levels when compared to the placebo [87]. In patients with metabolic syndrome, a whey protein intervention was equally efficient, since a significant reduction in TG levels (WMD: −17.12; 95% CI: −26.52, −7.72), total cholesterol (WMD: −10.88; 95% CI −18.60, −3.17), LDL-cholesterol levels (WMD: −8.47 95% CI: −16.59, −0.36), and total cholesterol/HDL cholesterol ratio (WMD: −0.26; 95% CI: −0.41, −0.10) was reported in a meta-analysis [76]. Another meta-analysis validated the beneficial effects of whey protein on TG serum levels, as a 12-week supplementation was associated with reduced triglyceride levels in comparison to a placebo (MD: −6.61, 95% CI: −11.06 to 2.17, I^2^: 70%, *p* < 0.01). The reductions in total cholesterol levels and LDL-c levels were observed only if the whey protein intervention was combined with exercise (MD: −8.58, −14.32 to −2.83, I^2^: 55%, *p* < 0.01), and this effect was achieved regardless of BMI when compared to the placebo [88]. Whey protein intake lowered statistically significant TG, total cholesterol, and LDL-c according to a large meta-analysis, while in obese/overweight, it decreased TG and total cholesterol, while elevating HDL-c in a statistically significant manner. The results indicated that whey protein supplementation is superior for lipid profile regulation in comparison to soy protein, milk protein, and casein [69]. These findings are in accordance with data observed in obese/overweight men, among which whey protein intake for 12 weeks lowered apo B lipoprotein, VLDL-c, LDL-c, TG, and total cholesterol, while upregulating the levels of apo A-I (*p* < 0.001) and HDL (*p* =0.001) relative to the baseline, and was the better treatment option when compared to soy protein [67]. In addition, a meta-analysis conducted in overweight/obese patients resulted in favorable HDL-c (MD = 0.42, 95% CI: 0.05–0.80, I^2^ 95%, P_heterogeneity_ < 0.01), and total cholesterol serum levels (MD = 0.53, 95% CI: 0.00–1.06, I^2^ = 89%, P_heterogeneity_ < 0.01) in the whey protein group, with no significant differences in TG or LDL cholesterol between the intervention and control groups [49]. Another meta-analysis conducted on obese/overweight patients, on the contrary, resulted in not only reduced TG values in whey protein treatment groups (−18.19, 95% CI: 22.49, −15.30; *p* < 0.001, I^2^ 100%, P_heterogeneity_ < 0.001) but also in an unfavorable decline in HDL levels in intervention groups vs. control groups (−6.07, 95% CI: 7.53, −4.61; *p* ≤0.001, I^2^ 100%, P_heterogeneity_ < 0.001) [53]. In specific vulnerable populations, such as older women, the favorable effect of whey protein on lipid status seemed intact. Preconditioned older women supplemented with whey proteins for 12 weeks in comparison to placebo had their total cholesterol/high density lipoprotein cholesterol ratio significantly improved [57]. The dose of whey proteins and supplementation interval seemed to affect the results. This was confirmed in a meta-analysis, as the beneficial effects of the whey protein intervention disappeared if a low supplemental whey dose was applied, in participants with low BMI, or under exercise training/energy restriction during the trial. However, the same meta-analysis indicated that whey protein supplementation significantly decreased TG levels by 0.11 mmol/L (95% CI: −0.21, 0 mmol/L), while total cholesterol levels, HDL-c, and LDL-c were not affected when the overall data were observed without subgroups [89]. Finally, perhaps the most significant effect of whey protein supplementation was observed in obese female patients, where even a short, four-week intervention led not only to reductions in fasting TG and total cholesterol levels, but also in a statistically significant decrease in intrahepatocellular lipid content, as measured by magnetic resonance spectroscopy. Notably, the use of a higher whey protein dose, 60 g/day, compared to the 40 g/day more commonly applied in most clinical trials, may represent a critical factor in enhancing the therapeutic potential of whey protein [90].

## 8. Whey Proteins and Liver Health

The serum levels of ALT and AST are often measured in order to check liver health and function. Elevated concentrations suggest an increased risk of liver injury. Apart from certain medications and alcohol abuse, some conditions including MASLD could be reflected through increased serum ALT and AST levels. Based on a large follow-up study that included more than 340,000 adults, up to 36% of MASLD participants had abnormal liver enzyme levels. Moreover, abnormal liver enzymes were more common in obese participants (BMI > 25 kg/m^2^) when compared to other BMI groups with MASLD (*p* < 0.001). In addition, increased liver enzyme levels were also associated with higher risk of DM, hypertension, dyslipidemia, and metabolic syndrome in MASLD individuals, with an odds ratio ranging from 1.23 to 6.44 in comparison to individuals with normal AST and ALT values without steatotic liver disease (all *p* values below 0.001). In addition, MASLD individuals with abnormal liver enzyme levels had a significantly higher risk of cardiovascular- and DM-related mortality (*p* < 0.001) [91]. Apart from AST and ALT, increased fat accumulation provokes the production of reactive oxygen species (ROS), resulting in cellular damage, inflammation, and fibrosis. Hence, oxidative stress and inflammation are closely linked with MASLD pathophysiology and progression. Among various cytokines and chemokines, tumor necrosis factor-alpha (TNF-α) and interleukin-6 (IL-6) are recognized as crucial mediators associated with hepatic inflammation and steatosis [92]. IL-6 drives the secretion of CRP that boosts the inflammatory response and systematic effects, resulting in steatohepatitis, advanced liver fibrosis, cardiovascular disease, and even mortality [93]. In a recent cross-sectional study that involved 20,141 adults of both genders, increased hs-CRP levels were marked in MASLD individuals of both genders. However, a greater increase in hs-CRP levels was observed in women compared to men [94].

Human studies on liver function following whey protein supplementation are still scarce and limited. Although whey protein supplementation had no effects on inflammatory markers (CRP, IL-6 and TNF-α) in trials conducted on a small number of volunteers [54,55,56,57,65], a large Korean epidemiological study that involved 5171 adults (aged 40–69) revealed that higher dairy protein consumption was inversely associated with the risk of NAFLD incidence in both genders older than 50 years [95]. Moreover, a decline in CRP values was observed in 95% of COVID-19 obese patients (BMI ≥ 30) supplemented with whey proteins [96]. In addition, in healthy men consuming whey protein supplementation (60 g/day) in conjunction with resistance exercise, a significant reduction in ALT by 41.0% and AST by 23.2% was observed after four weeks of intervention (*p* = 0.002 and *p* < 0.001, respectively) [97]. Beneficial effects of whey protein supplementation on liver health were also demonstrated in a clinical trial involving 38 patients of both genders diagnosed with NASH. Daily consumption of 20 g of whey protein isolate over 12 weeks led to a significant reduction in hepatic macrovesicular steatosis (*p* = 0.046), as well as in serum ALT (*p* = 0.016) and AST (*p* = 0.047) levels. Additionally, an improved antioxidative status was observed, indicated by increased plasma glutathione levels and total antioxidant capacity (all *p*-values below 0.05). These findings suggest that cysteine-rich whey protein isolate may prevent the progression of liver injury in patients with NASH by enhancing endogenous antioxidant defense mechanisms [98]. The antioxidative and anti-inflammatory effects of whey protein isolate (ProLYOtin^®^) were also evaluated in overweight adults with type 2 DM or impaired fasting glucose. Supplementation with 40 g/day of ProLYOtin^®^ resulted in an increase in glutathione peroxidase and the decrease in uric acid after 12 weeks, whereas no differences were observed in total antioxidant status and the levels of glutathione reductase. However, the reduction in uric acid suggests an oxidative status improvement, while the enhanced synthesis of a selenoprotein, gluthathione peroxidase, although not expected, was probably due to the higher intake of the selenium-rich whey protein isolate [54]. Despite the variability in the reported effects of whey protein on liver function across clinical studies, a reduction in ALT activity in women with PCOS was also recorded. Although no significant differences were observed, the obtained results suggested that whey proteins might potentially reduce the risk of MASLD development in women with PCOS [78].

Even if it is not yet clear whether whey protein supplementation can improve liver health, good tolerance was recorded, without serious adverse effects, among individuals with nonalcoholic steatohepatitis [98]. Moreover, its consumption is rarely associated with the onset of drug-induced liver injury [99]. However, the results obtained in pre-clinical studies are controversial [25]. Increased levels of AST, ALT, and inflammatory markers, as well as oxidative stress were reported in a limited number of animal studies in sedentary conditions [100,101,102]. Another point for consideration regarding safety should be the effect of a higher protein intake on kidney function. An increased glomerular filtration rate and elevated calcium excretion were observed as a consequence of whey protein intake in humans [103,104]. Further studies are needed to evaluate the effects of whey proteins on both AST and ALT, and inflammatory and oxidative stress markers, as well as kidney function parameters.

## 9. Conclusions and Future Perspectives

Despite the limited evidence obtained in clinical studies, this review provides insight into the beneficial effects of whey proteins on a spectrum of MASLD-related manifestation. Weight reduction, glucose and lipid metabolism improvements, blood pressure lowering effects, as well as reduced liver enzyme activity and oxidative stress were obtained in humans after whey protein supplementation. However, until now, there have been no studies that comprehensively examined the effects of whey proteins in patients with MASLD. Currently, lifestyle modifications such as diet and exercise remain the standard approach to MASLD treatment. Pharmacological options are also limited, as current treatments primarily target hypertension and insulin resistance as commonly related metabolic issues. Hence, whey proteins, as a natural, accessible dairy by-product rich in essential and sulfur-containing amino-acids, possess promising properties required for MASLD prevention and management. Well-designed cohort and clinical trials are required for further validation of the effects of whey proteins on liver health. Apart from the dose and duration of intervention, the type of whey protein supplementation in the context of animal species and whey production processes (i.e., concentrate, isolate, and hydrolysate) has to be optimized for achieving the best results. In addition, the safety of whey products during long-term intake is still unexplored. The impact of whey protein supplementation on microbiome species and associated biomarkers of disease also requires comprehensive understanding, having in mind that dysregulated gut microbiota have been recently linked with the onset and progression of MASLD. Therefore, there is an imperative need for examination of the interplay between whey proteins and bioactive peptides, obesity, hypertension, metabolic biomarkers, and liver injury.

## Figures and Tables

**Table 1:** Heterogeneity of the MASLD pathophysiology.

Phenotype	Clinical Characteristics
MASLD with a strong hepatic genetic component	− or + Insulin resistance ^1^
	− Adiposity
	− Dyslipidemia
	+ Type 2 diabetes
	− or +Cardiovascular disease ^2^
	+ or ++ MASH
	+ or ++ Fibrosis
MASLD with a strong metabolic component related to hepatic de novo lipogenesis	++ Insulin resistance
	++ Adiposity
	+++ Dyslipidemia
	++ Type 2 diabetes
	++ Cardiovascular disease
	+ MASH
	+ Fibrosis
MASLD with a strong metabolic component related to adipose tissue dysfunction	++ Insulin resistance
	− Adiposity
	+ Dyslipidemia
	++ Type 2 diabetes
	++ Cardiovascular disease
	+ MASH
	+ Fibrosis

^1^ − = moderate decrease or no change in prevalence or risk of disease; + = moderate increase; ++ = strong increase; +++ = very strong increase in prevalence or risk of disease; ^2^ obesity related.
